# Supplementary material for: Nudging individuals’ creativity using social labeling
Source: PLoS One. 2020 Feb 13;15(2):e0228961. doi: 10.1371/journal.pone.0228961 (PMC7018064; doi:10.1371/journal.pone.0228961)
Supplement: Attachment — Submitted filename: Response to the reviewers.docx [file pone.0228961.s005.docx]

**Nudging individuals’ creativity using social labeling: an experimental study**

Manuscript: PONE-D-19-16350

**Reviewer #1**

Dear Reviewer #1,

We sincerely thank you for your very detailed and relevant comments on our manuscript.

To facilitate your review of our answers and changes, we discuss your comments and explain how we have integrated each of them into this new version of our manuscript directly below each comment. Your original comments are reproduced in bold; our responses follow each comment; the modified text appearing in the new version of our manuscript is copied in blue.

**Minor: Study 2 & 3 need to be better described in the abstract. Second to the last sentence is not clear.**

We agree with this observation. We have rewritten the ending of our abstract in light of your comment:

Simple instructions have been shown to robustly influence individual creativity, which is key to solve local problems. Building on social labeling theory, we examine the possibility of nudging individual’s creativity using “creative” and “not creative” labels. Study 1 showed that subjects labeled as “creative” or “not creative” performed better in a creative task than unlabeled subjects and established the moderating effect of self-perceived creativity. Among subjects scoring low on self-perceived creativity, those labeled as “creative” performed better than those labeled as “not creative”. Conversely, among subjects scoring high on self-perceived creativity, those labeled as “not creative” tend to perform better than those labeled as “creative”. **Study 2 and Study 3 further explored the psychological mechanisms at play in both cases: specifically, Study 2 showed that applying a “creative” label has the ability to increase creative self-efficacy through self-perceived creativity, whereas Study 3 demonstrated that applying a “not creative” label has the ability to increase individual creativity performance through a higher involvement in the creative task.**

**Study 1: Please report the sample and the conditions that you included in the moderation analysis.**

We added the sample and the conditions that we included in the moderation analysis that we present for Study 1, as follows:

Going further, to test whether self-perceived creativity moderates the influence of a social label marking someone out **as creative (or not creative)** on individual creativity performance, we conducted a floodlight analysis using the procedure recommended by Cadario and Parguel (2014). H**alf of the participants were considered in this analysis (50 participants labeled as “creative” and 50 participants labeled as “not creative”).** Hayes' (2012) PROCESS macro (model 1) and 5000 bootstrapped samples were used to determine whether this moderating effect was significant. **The manipulation (i.e. a “not creative” label vs. a “creative” label) was included as the independent variable**, and individual creativity performance was included as the dependent variable.

**Please remove analysis with dichotomization on page 16, but report the figure directly from PROCESS Model 1 with a continuous variable. To do so, you need to add in the PROCESS the option: “generate data for the plot” (the same relevant for Study 3). Please report conditional effects in Model 1, rather than re-running the analysis in ANOVA**

We got rid of the analysis, which was based on dichotomization, and followed your recommendation. The new version of our manuscript now provides conditional effects and a plot directly generated from PROCESS. See below how we display both:

Further conditional analyses show that the “creative” (vs. “not creative”) label enhanced individual creativity performance (β=.46, t=2.13, p<.05) among participants scoring relatively low on self-perceived creativity (score of 4 out of 7), but that it only marginally reduced it (β=-.31, t=.23, p<.10) among those scoring relatively high (score of 6.5 out of 7). Corroborating H3a but only marginally H3b, these results are depicted in Figure 4.

Fig. 4 – Moderating effect of self-perceived creativity

**Study 2: Please clarify whether you measured creative self-efficacy, before and after exposing people to labels.**

We agree that the description of the procedure of Study 2 could be more precised and have improved it as follows:

Study 2 builds on Study 1 procedure. We first collected self-perceived creativity as a fake basis to ensure effective labeling of participants as “creative”, and participants had to register their age, gender and education level. Participants were then randomly assigned to one of the two conditions: a control condition vs. a “creative” label (as manipulated in Study 1). **After being randomly exposed to one of the two conditions, they were interrogated about their current self-perceived creativity and about the creative self-efficacy they would feel if they were now confronted with a creative task.** To conclude, we asked the participants who were labeled to recall the nature of the label they were provided with to check the validity of our manipulation, verified that no participant correctly guessed the purpose of the study and informed them that the label was purely randomly applied.

**Did you control for pre-labeled self-perceived creativity in the mediation analysis?**

We are a little bit confused. In actual fact, we controlled for pre-labeled self-perceived creativity in the mediation analysis in the first version of our manuscript. But to answer one of your concern about this first version, we finally got rid of our control variables in the second version, while adding the following sentence under Table 2: “Results do not change when controlling for participants’ … pre-label self-perceived creativity” (in yellow below).

Hence, controlling or not for pre-label self-perceived creativity does not change the analysis. Would you like us to go back to the first version, we would reintroduce it.

The same goes for the results of Study 3.

**Could you please provide a table that summarizes correlations between the variables in this study. The table will help to illustrate whether there is a collinearity problem between measures. The table should include pre-labeled self-perceived creativity, post-labeled self-perceived creativity, pre-(?) and post-self-efficacy, and study conditions.**

We did not measure pre-labeled self-efficacy to avoid any demand effect.

We would like to note that the previous version of our manuscript mentioned the correlation between pre- and post-label self-perceived creativity as follows:

Our measures proved to be reliable: Cronbach’s α of 894 for the items measuring pre-label self-perceived creativity, Cronbach’s α of 945 for the items measuring creative self-efficacy and correlation of .794 for the two items measuring post-label self-perceived creativity. **Of note, the measures of pre- and post-label self-perceived creativity are correlated at .562.**

In the new version, we display the full correlation table you asked for, as follows:

Our measures proved to be reliable: Cronbach’s α of 894 for the items measuring pre-label self-perceived creativity, Cronbach’s α of 945 for the items measuring creative self-efficacy and correlation of .794 for the two items measuring post-label self-perceived creativity. **Table 2 displays the correlation between all the creative self-perceptions for the two conditions.**

|  |  | Pre-label self-perceived creativity | Post-label self-perceived creativity | Post-label creative self-efficacy |
| --- | --- | --- | --- | --- |
| No label | Pre-label self-perceived creativity | 1 | .514 | .530 |
|  | Post-label self-perceived creativity |  | 1 | .733 |
| “Creative” label | Pre-label self-perceived creativity | 1 | .624 | .713 |
|  | Post-label self-perceived creativity |  | 1 | .822 |

As already proposed in our response to your previous comment, we can go back to our first version and re-introduce our control variables.

**I recommend reporting this study with repeated measure design, in which you have pre and post-self-perceived creativity with conditions as a factor. (See my comment below)**

We analyzed the influence of pre-label self-perceived creativity and pre-label self-perceived creativity using a repeated measure design and found a significant interaction effect of time of measurement and our manipulation. Your suggestion would thus work, but we do not agree on this point for two reasons.

First, from a conceptual point of view, Study 2 and Study 3 have the objective of exploring potential underlying mechanisms behind the effect of the label as it was found in Study 1. Study 2 compares the “Creative” label condition with a no label condition, Study 3 compares the “Not creative” label condition with a no label condition. Hence, we do not explore anymore the moderating effect of Study 1, which compared the “Creative” and the “Not creative” conditions. Hence, we actually need mediation analyses both in Study 2 and in Study 3, and not a repeated measure design. In our hypotheses, self-perceived creativity is not the dependent variable but the mediating variable and a repeated measure design could not allow testing the mediation we are focusing on. Hence, to keep it simpler, we kept our mediation analysis using PROCESS.

That said, we realized that we may have not been clear on the structuration or our data collections and may therefore have triggered your comment about moderation by the additional analyses we made in the previous version of our paper. We apologize for that.

In the new version, we tried to make it clearer in the abstract

Simple instructions have been shown to robustly influence individual creativity, which is key to solve local problems. Building on social labeling theory, we examine the possibility of nudging individual’s creativity using “creative” and “not creative” labels. Study 1 showed that subjects labeled as “creative” or “not creative” performed better in a creative task than unlabeled subjects and established the moderating effect of self-perceived creativity. Among subjects scoring low on self-perceived creativity, those labeled as “creative” performed better than those labeled as “not creative”. Conversely, among subjects scoring high on self-perceived creativity, those labeled as “not creative” tend to perform better than those labeled as “creative”. **Study 2 and Study 3 further explored the psychological mechanisms at play in both cases: specifically, Study 2 showed that applying a “creative” label has the ability to increase creative self-efficacy through self-perceived creativity, whereas Study 3 demonstrated that applying a “not creative” label has the ability to increase individual creativity performance through a higher involvement in the creative task.**

And also, at the end of Study 1 as follows:

Study 2 and Study 3 extend these first findings on the impact of social labeling in creativity management by investigating the psychological **mediating mechanisms** at play when applicating a “creative” label (Study 2) or a “not creative” label (Study 3), **versus no label**.

Then, for each of these two studies, we now provide the figure of the mediation model we are interested in. We really thank you for having pointed out this weakness, which imbued the flow of our rationale in the previous version of our manuscript.

Regarding Study 2:

Fig. 5 – Mediating mechanism at play to explain the “creative” label effect

Regarding Study 3:

Fig. 6 – Mediating mechanisms at play to explain the “not creative” label effect

Of note, the model we present now to explain the “not creative” label effect is totally based on your invitation to integrate the brick-task performance in our analyses. We have to confess that we did not do it at the previous round for several reasons 1/ this task was a pretext to address mediating mechanism at play, we are actually less familiar with its use than with the egg task (this is actually why we set no minimal time to complete the brick task) and 2/ we ran out of time.

Now, we have followed your suggestion and are very happy to be able to display a more interesting model following your comment and would like to thank you for this valuable opportunity you gave us to improve our paper.

**Study 3: The analysis of study 3 is confusing. It needs a picture with a “classic” triangle with a mediator on top. The conclusion as it stands now “both significantly damages post-label self-perceived creativity (F=12.19, p<.01, ηp2=.057, ω=0.97)” looks like a result of ANOVA analysis but not Hayes Model 4. Please re-run the analysis as I suggested below.**

We followed your suggestions by adding a triangle figure (see our answer to your previous comment above) and by running Hayes Model 4 instead of ANOVA analysis.

The aim of Study 3 is to examine whether self-perceived creativity and the involvement in the creative task mediate the influence of a “not creative” label on individual performance creativity. To do so, we used Hayes' (2012) PROCESS macro (model 4) and 1000 bootstrapped samples. The analysis first shows that the application of the label significantly reduces post-label self-perceived creativity (β=-.59, p<.01, ηp2=.059, ω=0.92) and increases time spent on the task (β=51.77, p<.05, ηp2=.018, ω=0.43). Following, both post-label self-perceived creativity (β=.34, p<.05) and time spent on the task (β=.0058, p<.01) transfer to individual performance creativity. A bias-corrected 90% confidence interval for the indirect effect ranges from -0.42 to -0.02, with a point estimate of -0.20 for post-label self-perceived creativity and from 0.04 to 0.56, with a point estimate of 0.30 for the time spent on the task. Since the confidence interval does not include 0, this pattern provides evidence of a marginally significant indirect effect on individual performance creativity: the “not creative” label reduces self-perceived creativity but enhances the involvement in the creative task, which – in turn – modifies individual performance creativity. This mediation is partial as the direct effect of the “not creative” label on individual performance creativity is also marginally significant (β=.76, p<.10). Table 4 displays the results of this analysis.

Table 4 – Mediation analysis

|  | **Post label self-perceived creativity** | **Involvement in the creative task** | **Individual performance creativity** |
| --- | --- | --- | --- |
| No label vs. “Not creative” label | **-.59***** | **51,77**** |  |
| Post-label Self-perceived creativity |  |  | **.34**** |
| Involvement in the creative task |  |  | **.0058***** |
| Direct effect of the label on individual performance creativity IC (90%) | | | **[.02;1.51]** |
| Indirect effect via post-label self-perceived creativity IC (90%) | | | **[-.42;-.02]** |
| Indirect effect via the involvement in the creative task IC (90%) | | | **[.04;.56]** |

*** *p* < .01, ** *p* < .05, * *p* < .10. Of note, when controlling for participants’ pre-label self-perceived creativity, the indirect effect via post-label self-perceived creativity is no more significant.

**Study 2 & 3 analysis and results are problematic as they stand now. Please state at the beginning that you are planning to test in two different studies conditions with creative and non-creative labels versus control conditions.**

We have now stressed this point even more clearly before introducing Studies 1, 2 and 3.

We will test our conceptual model in Study 1, before further exploring the psychological mechanisms at play when labeling an individual as “creative” in Study 2, i.e. the activation of creative self-efficacy, or when labeling an individual as “not creative” in Study 3, i.e. an enhanced involvement in the creative task. We will therefore test in two follow-up studies why both “creative” and “not creative” labels may enhance individual creativity performance.

**Both studies need to have identical analyses answering three questions.**

Study 2 and Study 3 display now identical mediation analyses. Study 1 has a different status, hence relies on moderation analyses.

**Question 1: whether a label changes self-perceived creativity before and after the manipulation. This could be accomplished with a repeated measure design and the conditions as a factor.**

As we explained it in response to your earlier comment, regarding Study 2, we interrogate mediation effect. Please note that the moderating effect shown in Study 1 is based on the comparison between a “creative” and a “not creative” conditions. As Study 2 and Study 3 do not rely on the same comparisons (i.e., they compare the “ creative” condition with the control condition, or the “not creative” condition with the control condition), they have not the ambition to replicate the first moderating effect.

**Question 2: whether low base self-perceived creativity moderates the effect of conditions on behavior (or its proxy, self-efficacy). In both studies, Hayes model 1 needs to be applied to answer this question. In model 1, conditions need to be included as independent variables, while base self-perception as moderator and dependent variables should be: self-efficacy (a proxy of behavior) in study 2 and a brick-task performance in Study 3. This analysis needs figures reported from Model 1.**

As we explained it above, we do not focus in Study 2 and Study 3 on the moderation but on the mediation. We want to know why respondents are influenced by labels.

However, regarding your invitation to include the individual creativity performance at the brick task, we strictly followed it, as you can see in the section dedicated to Study 3. Again, many thanks for this excellent idea.

**Question 3: whether the change in self-perceived creativity influences participants’ behavior (or a proxy of it). A mediation analysis should be used. For study 2: conditions (independent variable)->post-self-perception controlling for pre-self perception (mediator) -> self-efficacy (dependent variable). For study 3: conditions (independent variable)->post-self perception controlling for pre-self perception (mediator) -> brick-task performance (dependent variable). This analysis needs figures.**

We introduced these analyses and the corresponding figures (see above).

**Minor comment: please keep boosting samples consistent throughout the manuscript; report statistics with degrees of freedom e.g. t (df?)=1.98, p<.05; and confidence intervals.**

We corrected the problems related to degrees of freedom and checked that confidence intervals were indicated were needed.

**In study 1, you suggested “to make our label credible, participants were first asked to complete an initial questionnaire about their self-assessed ability to generate creative ideas” is it the same as “pre-label self-perceived creativity measure” in studies 2&3? If no, how did you mask a persuasive intend in study 2&3?**

It is the same initial questionnaire about the self-assessed ability to generate creative ideas, in all three studies, that is then also used in all three studies to measure pre-label self-perceived creativity. We have consistently indicated in the “Measures” sections of the three studies that we measure pre-label self-perceived creativity as such.

**About the last comment: please do not dichotomize variables but report the statistics and figures from Hayes directly.**

We corrected this problem.

**Editor**

Dear Valerio,

We sincerely thank you for this new opportunity to improve our manuscript. We answered each Reviewer 1’s comments in the previous pages.

Still, you made the same comments in your letter as in the previous round. So we do not know whether you found our answers. To make sure, we copy paste our previous answers below. To facilitate your review, we discuss your comments and explain how we have integrated each of them into this new version of our manuscript directly below each comment. Your original comments are reproduced in bold; our responses follow each comment; the modified text appearing in the new version of our manuscript is copied in blue.

**Particular attention, however, should be given to the issue raised by both reviewers regarding the smallness of the sample (especially in light of the current replicability crisis) (…)**

We collected new data for two reasons.

First, we did so to address Reviewers’ comments regarding the smallness of our samples. Precisely, we added 26 respondents in what appeared as Study 1 in the first version of our submission (which is now Study 2). In the new version of our manuscript, we now display high-powered test regarding the influence of the “creative” label, but mostly medium-powered tests regarding the influence of the “not creative” label. As we found that this symmetrical influence was clearly of interest, we chose to keep it in the new version of our manuscript and acknowledge this in our final discussion. However, would you prefer not to keep the focus on the “not creative” label in the manuscript, we would obviously follow your advice.

Second, we collected new data (N=202) to test the relevance of the psychological mechanism at play in the influence of the “not creative” label. Precisely, we made a whole new data collection, appearing as Study 3 in the new version of the manuscript, to back our hypothesis regarding resistance to the negative label to restore individuals’ self-esteem. And, Study 3 actually provides interesting empirical evidence of the fact that subjects labeled as “not creative” react to the label by investing more efforts in the creative task.

The databases that are provided along with this new submission include the fresh data collected.

**(…) and to the issue raised by Reviewer 1 regarding deception (please explain exactly which parts of the experiments involved deception and why you think this is not a problem for your experiments).**

We designed our experiment as a deception experiment and also perfectly right mentioning that deception is a big issue in experimental economics. As mentioned by Tyler and Amodio in 2015 the *Oxford Handbook of Experimental Economic Methodology*, “there is no subject that provokes so much emotion among economists as the use of deception in an experiment”.

Tyler, T. R., & Amodio, D. M. (2015). Psychology and economics: areas of convergence and difference. Handbook of experimental economic methodology. Oxford University Press, Oxford, 181-196.

Still, deception experiments are more than frequent in psychology experiments and have been regarded as legitimate by many social sciences that have been using experiments for decades.

Deception experiments are not used by psychologist for amoral reasons, but to ensure internal validity. Precisely, our manipulation allows to draw conclusion that could not be drawn otherwise. We cannot imagine that it would have been possible to tell our subjects, as an introduction of the experiment, that we were about to put them in different fake conditions regarding their personal creativity. Such a disclosure would clearly have modified the way our subjects would have presented themselves and the way they would have responded to our label.

Tyler and Amodio (2015) identify a specific problem with deception experiments as they can “spoil the subject pool” for subsequent experiments. Though this argument could be relevant when carrying experiments using internal student samples, it is less relevant when considering the external sample of a market research institute. As a matter of fact, we have been using the same market research institute for years and always ask our subjects what they thought was the actual objective of the study they had just participated in and none of them (in all our data collections) correctly guessed it. Following, we are pretty confident on the fact that deception experiments conducted on very large pool of respondents and not on very restricted local pool do not “spoil the sample”.

Another problem is more moral and regards the fact to put subjects in scenario that make them believe for few minutes that they are not creative. To circumvent this problem, we used a clear debriefing statement at the end of the experiment to make clear that all subjects are. As an addition, we would also like to mention that all subjects participating in our studies gave an active consent before participating and that our protocols were all validated by one of the researchers’ IRB.

As written in the first version of our manuscript:

For ethical purposes, after completing the whole procedure, participants were informed in the following words that the label was purely randomly applied: “Your creativity score stated at the start of the survey was randomly assigned for the purpose of our research. It does not reflect your actual level of creativity at all.” As for Study 2 and Study 3, Study 1 experimental procedure was approved by the first author’s Institutional Review Board.

In the first revised version (R1), we also added a specific comment on deception (see the sentence in bold characters in the previous paragraph) to fully address this issue.

Of note, we acknowledge that disguising what we were studying deceives subjects but appears necessary to preserve the internal validity of our results. Though it can be considered as a big issue by economists, most social sciences that have been carrying experiments for decades tend to consider it as a legitimate procedure (Tyler & Amodio, 2015).

**Moreover, I would like to mention that social labels have been recently used also to impact people's decisions in economic games**

**(https://www.sciencedirect.com/science/article/pii/S0022103116302098; https://www.sciencedirect.com/science/article/pii/S0022103118302841; http://journal.sjdm.org/17/171107/jdm171107.pdf;** [**http://journal.sjdm.org/19/190107/jdm190107.pdf**](http://journal.sjdm.org/19/190107/jdm190107.pdf)**).**

**Note that I am the author of some of these papers. My decision on this manuscript will obviously be independent of whether you will decide to include or not these papers in your reference list. I have just thought that you might find them relevant.**

We thank you for the references on “labels as framing” you mentioned in your review synthesis. We discussed their opportunity but finally decided not to introduce them in our manuscript as we envision the social labeling theory as a type of self-perception theory, whereas framing more often applies to the environment design in which the subjects form attitudes and decisions. Still, we really appreciated to learn more on the way experimental economists integrate psychological constructs to advance social sciences at large.
