# Supplementary material for: Nudging individuals’ creativity using social labeling
Source: PLoS One. 2020 Feb 13;15(2):e0228961. doi: 10.1371/journal.pone.0228961 (PMC7018064; doi:10.1371/journal.pone.0228961)
Supplement: Attachment — Submitted filename: Response to the reviewers.docx [file pone.0228961.s006.docx]

**Nudging individuals’ creativity using social labeling: an experimental study**

Manuscript: PONE-D-19-16350

**Reviewer #1**

Dear Reviewer #1,

We sincerely thank you for your new relevant comments on our manuscript. AT that stage, it is really time to sincerely thank you for having led us to this level of methodological rigor.

To facilitate your review of our answers and changes, we discuss your comments and explain how we have integrated each of them into this new version of our manuscript directly below each comment. Your original comments are reproduced in bold; our responses follow each comment; the modified text appearing in the new version of our manuscript is copied in blue.

**I assume that in Figure 3, error bars show the standard deviation. The figure should show a standard error, it allows readers to estimate the statistical difference from the figure.**

You are perfectly right. We corrected this following your suggestion. This is how it now appears in the new version of our manuscript:

Standard errors are presented as bars directly on the graphic, and standard deviations have been added on a specific Table right under the graphic.

**Confidence interval 90 is unusual, the conventional way to report a confidence interval of 95 as it corresponds to conventional expectations of p < .05. It is concerning that when you add pre-label self-perceived creativity in study 3 the indirect effect disappears. However, it could be an issue of low power. Can you please comment on the effect sizes, e.g. what is the mean difference between conditions in post-label self-creativity? and what is the mean difference between conditions in individual creativity performance? That information will allow a reader to estimate the magnitude of the effect of the "non-creative" label.**

We acknowledge that “proper inference requires full reporting and transparency” (Wasserstein & Lazar, 2016). To answer your comment, we first introduced the means in individual creativity performance, post-label creativity, and time spent on the creative task for the different conditions we manipulate. This is how it now appears for Study 1 and Study 2:

Study 1

Further conditional analyses show that the “creative” (vs. “not creative”) label **enhanced individual creativity performance (6.3 vs. 5.2, β=.46, t93=2.13, p<.05) among the 18 participants** scoring relatively low on self-perceived creativity (score under 4 out of 7). It **marginally reduced it (5.8 vs. 6.0, β=-.31, t93=1.38, p<.10) among the 18 participants** scoring relatively high on self-perceived creativity (score over 6.33 out of 7). Corroborating H3a but only marginally H3b, these results are depicted in Figure 4.

Study 3

The aim of Study 3 is to examine whether self-perceived creativity and the involvement in the creative task mediate the influence of a “not creative” label on individual performance creativity. To do so, we used Hayes' (2012) PROCESS macro (model 4) and 1000 bootstrapped samples. The analysis first shows that the application of the “not creative” label **significantly reduces post-label self-perceived creativity (4.4 vs. 4.9 in the “no label” condition, β=-.59, p<.01, η_p_^2^=.059, ω=0.92) and increases time spent on the task (138 vs. 93 seconds in the “no label” condition, β=51.77, p<.05, η_p_^2^=.018, ω=0.43).** Following, both post-label self-perceived creativity (β=.34, *p*<.05) and time spent on the task (β=.0058, *p*<.01) transfer to individual performance creativity, **which scores at 5.7 for the participants labeled as “not creative” vs. 5.1 for the unlabeled participants.**

Of note, to keep our writing consistent throughout the manuscript, we also changed the way we present the results for Study 2:

To do so, we used Hayes' (2012) PROCESS macro (model 4) and 1000 bootstrapped samples. The analysis first shows that the application of the label **significantly increases post-label self-perceived creativity (5.2 vs. 4.5 in the “no label” condition, β=.65, p<.01, η_p_^2^=.067, ω=0.85).** Following, post-label self-perceived creativity **transfers to creative self-efficacy (5.5 vs. 4.9 in the “no label” condition, β=.78, *p*<.01).**

For sure, these precisions will help future readers to evaluate the scope of our findings and we thank you for this valuable suggestion.

Going further, we want to note here that we already underlined the presence of “marginally significant” results in our general ending discussion as follows:

Still, we acknowledge here that the positive effect of a negative “not creative” label on individual creativity performance only appears marginal in both Study 1 and Study 3, and therefore calls for replication.

However, we mentioned it without really discussing it. The new version of our manuscript provides such a discussion.

Regarding Study 1’s case of marginal significance, which concerns the influence of a positive versus negative creative label among those who, initially, perceive themselves as highly creative, it is interesting to note that this marginal significance does not rule out the moderating effect we postulate in Hypothesis 1. In actual facts, this influence among those who, initially, perceive themselves as low in creativity is clearly significant. Besides, comparing the negative label versus the no label conditions, Study 3’s data plainly corroborate the effect that we found as only marginal when comparing the positive versus negative conditions in Study 1. This provides an “external” evidence for our theoretical proposition and enhances our confidence that it is a valuable one, though it deserves to be taken further by future research.

Regarding now Study 3’s case of marginal significance, it applies to the influence of the mediating variables and not to the focus dependant variable, namely participants’ individual creative performance. In this Study, as in Study 1, this focus dependent variable is of a behavioral (and not of a perceptual) nature, which is usually far more difficult to observe. This first finding is therefore of real interest for the literature developed in psychology of creativity.

In the new version of our manuscript, this is how we present this discussion:

Though demonstrating the significant positive effect of the “not creative” label on individual creativity performance, Study 1 and Study 3 also display specific marginal effects to be discussed. In Study 1, an insufficient sample size when testing the moderating influence of self-perceived creativity may be responsible for this result. Still, Study 3 has provided additional data showing that the application of a “not creative” label enhances subjects involvement in the creative task, but only among those scoring high on self-perceived creativity. This external evidence (not presented here to keep the paper consistent and reasonable in size) adds confidence in the moderating influence of self-perceived creativity in the case of the “not creative” label. In Study 3, the marginal effect we found applies to the mediation by time spent on the creative task. As behavioral effects are often more challenging to study, demonstrating the mediating role of subjects’ involvement in the influence of the “not creative” label on creative behavior already provides a relevant finding. In the end, the marginal effects we found in Study 1 and Study 3 do not undermine our general and pioneering contribution while still calling for further research to more finely understand social labeling techniques.

Again, we would like to highlight how grateful we are for all the constructive comments you made throughout the different rounds of this submission.

**References**

Wasserstein, R. L., & Lazar, N. A. (2016). The ASA statement on p-values: context, process, and purpose.
